# Training in Ultrasound to Determine Gestational Age in Low- and Middle- Income Countries: A Systematic Review

**DOI:** 10.3389/fgwh.2022.854198

**Published:** 2022-03-18

**Authors:** Alexandra C. Viner, Isioma D. Okolo, Jane E. Norman, Sarah J. Stock, Rebecca M. Reynolds

**Affiliations:** ^1^Medical Research Council (MRC) Centre for Reproductive Health, The University of Edinburgh, Edinburgh, United Kingdom; ^2^Program in Global Surgery and Social Change, Harvard Medical School, Boston, MA, United States; ^3^Faculty of Health Sciences, The University of Bristol, Bristol, United Kingdom; ^4^Usher Institute, The University of Edinburgh, Edinburgh, United Kingdom; ^5^Centre for Cardiovascular Science, The University of Edinburgh, Edinburgh, United Kingdom

**Keywords:** training, gestational age, ultrasound, low-income, middle-income

## Abstract

**Introduction:**

Establishing an accurate gestational age is essential for the optimum management of pregnancy, delivery and neonatal care, with improved estimates of gestational age considered a public health priority by the World Health Organization (WHO). Although ultrasound is considered the most precise method to achieve this, it is unavailable to many women in low- and middle- income countries (LMICs), where the lack of trained practitioners is considered a major barrier. This systematic review explores what initiatives have previously been undertaken to train staff to date pregnancies using ultrasound, which were successful and what barriers and facilitators influenced training.

**Methods:**

The systematic review was conducted according to PRISMA guidelines and the protocol registered (PROSPERO CRD42019154619). Searches were last performed in July 2021. Studies were screened independently by two assessors, with data extracted by one and verified by the other. Both reviewers graded the methodological quality using the Mixed Methods Assessment Tool. Results were collated within prespecified domains, generating a narrative synthesis.

**Results:**

25/1,262 studies were eligible for inclusion, all of which were programme evaluations. Eighteen were undertaken in Africa, three in South-East Asia, one in South America, and three across multiple sites, including those in Africa, Asia, and South America. Five programs specified criteria to pass, and within these 96% of trainees did so. Trainee follow up was undertaken in 18 studies. Ten met recommendations for training outlined by the International Society of Ultrasound in Obstetrics and Gynecology (ISUOG) but only 1 met the current standards set by the WHO.

**Discussion:**

This systematic review is the first to evaluate this topic and has uncovered major inconsistencies in the delivery and reporting of basic obstetric ultrasound training in LMICs, with the majority of programs not meeting minimum recommendations. By identifying these issues, we have highlighted key areas for improvement and made recommendations for reporting according to the RE-AIM framework. With an increasing focus on the importance of improving estimates of gestational age in LMICs, we believe these findings will be of significance to those seeking to develop and expand the provision of sustainable obstetric ultrasound in LMICs.

**Systematic Review Registration:**

https://www.crd.york.ac.uk/prospero/display_record.php?ID=CRD42019154619, PROSPERO CRD42019154619.

## Introduction

Gestational age is the age of the fetus, from the 1st day of the last menstrual period to the current date, as given in weeks and days. Establishing an accurate gestational age is fundamental to the optimum management of pregnancy, delivery and neonatal care, as well as an essential component of strategies to improve neonatal outcome. Not only are precise estimates of gestational age required to facilitate a more accurate global reporting of preterm birth and intrauterine growth restriction, but also to permit vital ongoing research into how to improve outcomes for these babies. Indeed the World Health Organization (WHO) has regularly cited the need for improved estimates of gestational age as a public health priority (1–3). While accurate estimates of gestational age are important in any setting, they are even more so in low- and middle- income countries (LMICs), where the burden of perinatal complications is high, but the availability of resources and a contextualized evidence base low.

Although there are a number of different ways to determine gestational age, they vary in their accuracy, with early estimation using ultrasound considered the most accurate (4–7). However, despite recommendation from the WHO that all women receive an ultrasound scan prior to 24 weeks to “estimate gestational age, improve detection of fetal anomalies and multiple pregnancies and reduce induction of labor for post term pregnancy” (8) this remains unavailable to many women living in LMICs. Here, gestational age is derived from the last menstrual period or by abdominal palpation, both of which are less accurate than ultrasound. Scaled provision of ultrasound is challenging for multiple reasons (9–14), with the lack of trained practitioners considered a major barrier (11, 12).

While there is no universally agreed or standardized approach to training in ultrasound, the International Society of Ultrasound in Obstetrics and Gynecology (ISUOG) and WHO do both provide guidance. The ISUOG recommends that training should involve the combination of both didactic and “hands on” tuition, as well as practical assessment, with practitioners able to demonstrate adequate proficiency prior to independent practice (15). The WHO recommends that trainees undertake a minimum number of supervised scans (*n* = 50 1st trimester and *n* = 200 2nd/3rd trimester), although makes no specific reference as to what should constitute competency itself (16).

With an increasing focus on the importance of improving estimates of gestational age in LMICs, we believe it is important to establish the current evidence pertaining to previous initiatives to train staff in this context to date pregnancies using ultrasound. Therefore, the aim of this systematic review was to establish; what proportion of training was delivered in line with recommended standards, what proportion of initiatives were successful and what factors influenced the delivery of training.

## Methods

### Search Strategy

Our search strategy aimed to identify all available literature relating to any previous initiatives undertaken to train practitioners in the use of ultrasound to determine gestational age in LMICs. Following testing, searches were initially performed in November 2019 and updated in July 2021. Databases searched included EMBASE, AMED, MEDLINE, CINAHL, AIM, Global Health, Global Index Medicus, Cochrane, and Web of Science and we performed additional checks of the gray literature and reference lists of included papers to ensure additional relevant studies were not missed. The review was registered with the International prospective register of systematic reviews (PROSPERO Record CRD42019154619) and was conducted in accordance to the Preferred Reporting Items for Systematic Reviews and Meta-Analyses Protocols (PRISMA). The search strategy is available in the Supplementary Materials.

#### Population

Healthcare workers in LMICs.

#### Intervention

Training in ultrasound to determine gestational age.

#### Comparison

None.

#### Outcomes

- What proportion of training was delivered in line with recommended standards (ISUOG/WHO)
- What proportion of training was successful (trainees passed assessment)
- What factors influenced delivery of training.

### Inclusion Criteria

All reports or studies of any design where participants either provided or underwent training in ultrasound to determine gestational age in LMICs, as defined by the World Bank list of Economies (June 2020) (15), were included. There was no restriction placed on date, however abstracts must have been available in English to be considered for initial screening.

### Exclusion Criteria

**Figure 1:**
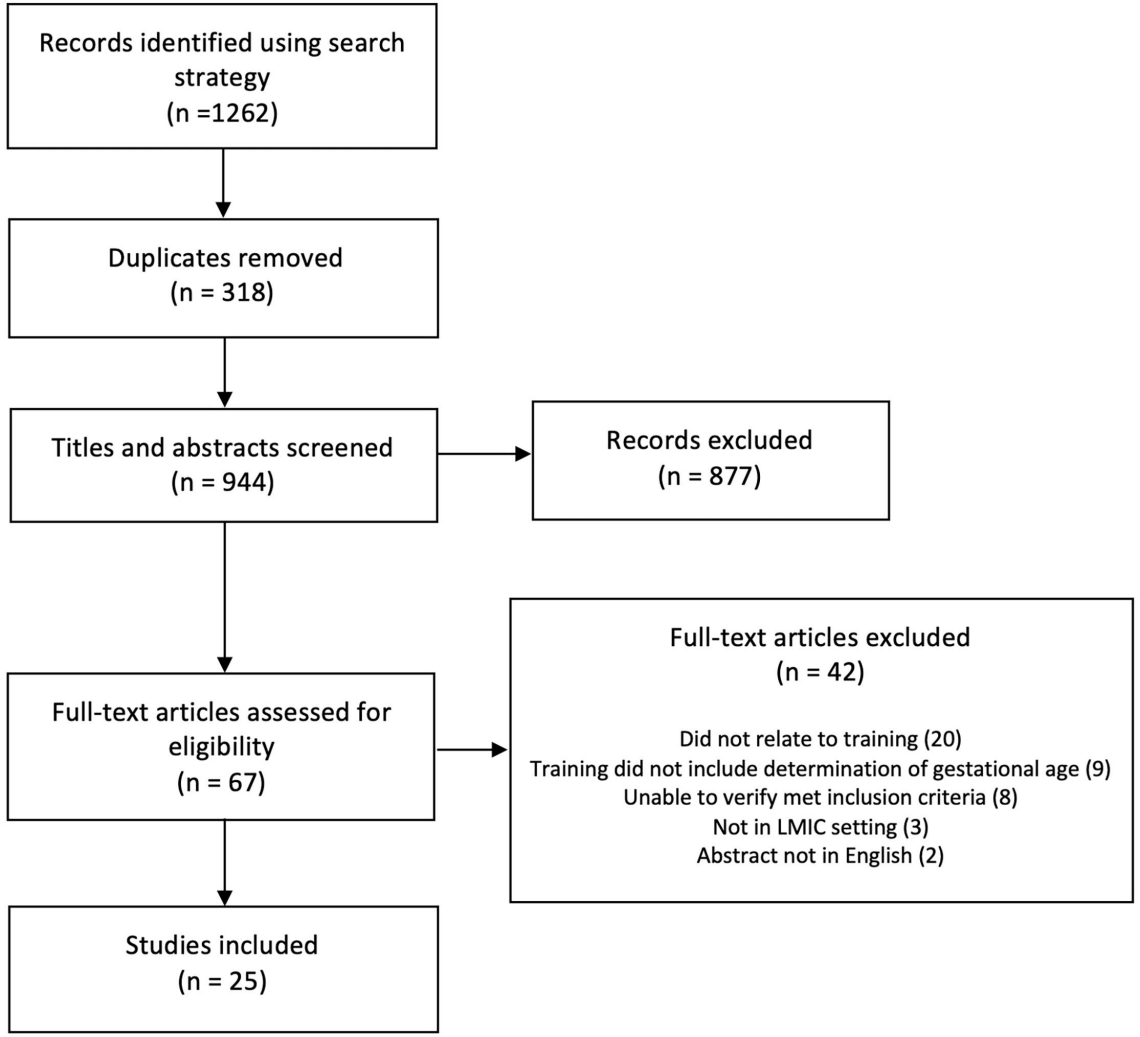
PRISMA diagram.

Studies which were not undertaken in LMICs were excluded, as were studies relating to training in obstetric ultrasound that did not include the determination of gestational age. Where there was uncertainty as to whether the assessment of gestational age was included in the training programs, further information was sought online and the authors contacted directly for clarification (*n* = 14) (16–29). If the inclusion of gestational age assessment in the training could not be verified, the studies were excluded (*n* = 9) (16, 17, 19–24, 30). See Figure 1 for further details.

### Study Selection and Data Extraction

Eligible abstracts were uploaded and managed in Covidence systematic review software (Veritas Health Innovation) and duplicates removed. All abstracts were screened independently by two assessors (AV and IO) according to the criteria outlined above. Data was extracted directly into a customized form created within Covidence by one reviewer (AV) and verified by the other (IO). Any disagreements were resolved by discussion, without the need for a third reviewer. Where multiple papers were identified pertaining to the same study or training programme, they were amalgamated into a single study. All were reviewed for data extraction, with the manuscript containing the majority of the information cited as the primary source.

### Study Quality Assessment

The methodological quality of the studies was assessed independently by two reviewers (AV and IO) using the Mixed Methods Assessment Toot (MMAT). The MMAT was selected to permit the simultaneous assessment of a range of different study types (32). In situations where the training had been undertaken as part of a larger study, only the evidence pertaining to the quality of the educational intervention itself was assessed, as opposed to the methodologies used for the “parent” study. Scores were not used to dictate inclusion or exclusion, rather to illustrate the quality of the evidence.

### Data Analysis

In the absence of data suitable for meta-analysis, results were collated according to the Cochrane Synthesis Without Meta-analysis (SWiM) guidelines (33). Data was extracted into pre-defined groups generated according to the PICO format. These included who participated in and delivered training, the setting and duration, what was taught within the curricula and how were trainees assessed and followed up. The overall quality of training was evaluated based on its adherence to ISUOG and WHO recommendations and where possible, success was defined as the proportion of trainees who passed. If formally reported in the context of implementation outcomes, data was also collected on factors identified as having facilitated or acted as a barrier for training.

## Results

In total, 25/1,262 studies were included (Figure 1) (27–30, 34–52) all of which were programme evaluations. The majority were undertaken in Africa (*n* = 18, 72%) (25–28, 30, 33, 35, 36, 38, 40–42, 45–48, 50), with three in South East Asia (12%) (31, 40, 45), one in South America (4%) (51), and several across multiple sites in Africa, Asia, and South America (*n* = 3, 12%) (35, 44, 53). Most were descriptive studies (*n* = 23, 92%) (27, 29, 30, 34–41, 43–54) of which 3 (12%) (40, 43, 46) employed mixed methods. The remaining 2 (8%) (28, 42) were case reports. All studies were published from 2008 onwards.

Just over half of the included studies (*n* = 15, 60%) (27, 29–31, 34, 36–38, 40–42, 47, 48, 50, 52) focused on evaluating the training programmes themselves, whereas the remainder (*n* = 10, 40%) (28, 35, 39, 43–46, 49, 51, 53) described training which had been undertaken as part of a larger study to facilitate a different research question. Of these, two provided training with the aim of developing new standards for fetal dating and growth (35, 53), three sought to assess the impact of introducing antenatal ultrasound on maternal and fetal outcomes (39, 44, 46), one evaluated the implementation of the INTERGROWTH-21st standards (43) and one evaluated a new tele-ultrasound system (51). In the remaining three studies, training was provided to ensure access to an accurately dated study cohort (28, 45, 49).

Four (16%) studies presented data on the barriers and facilitators to the provision of ultrasound training, having formally reported this in the context of implementation outcomes (40, 43, 46, 53). Supplementary Table 2 provides an overview of the main characteristics of each study.

### Quality Appraisal of the Included Papers

Two (8%) (42, 52) studies achieved the maximum MMAT score, with 9 (36%) (29, 30, 34, 37, 43–46, 49) rated as of moderate quality. Ten (40%) (25, 27, 28, 33, 36, 38–40, 47, 48) studies were considered low quality. The remaining 4 (16%) (26, 35, 41, 50) did not provide sufficient information to permit a full assessment.

### Participants and Faculty

Training was provided to local practitioners in all studies, with very few (*n* = 5, 20%) designed for those with previous experience of using ultrasound (27, 29, 35, 43, 53). Instead, over half (*n* = 14, 56%) focused on training those who had not used ultrasound before (28, 30, 31, 36, 38, 41, 42, 44–49, 51). Six (24%) of the studies did not comment on trainees' prior experience (34, 37, 39, 40, 50, 52). Of the 10 (40%) studies where training had been developed by local teams (29–31, 36, 39, 40, 42, 44, 52, 53), nearly all (8, 80%) were in collaboration with overseas institutions (30, 31, 36, 39, 40, 42, 44, 53). Local practitioners delivered the training in 14 (56%) of the programs (29–31, 36, 39, 40, 42–47, 52, 53).

### Setting and Duration of Training

Just over half of the studies (*n* = 15, 56%) (27, 28, 31, 34, 37, 38, 41, 43, 44, 46–51) reported training that had been delivered in a clinical setting, with the remainder undertaken across a variety of other sites. These included The Ernest Cook Ultrasound Research and Education Institute (ECUREI) in Kampala, Uganda, a specialist ultrasound training center (*n* = 3, 12%) (30, 36, 39), a refugee camp (*n* = 1, 4%) (31), a nursing school (*n* = 1, 4%) (42), and a conference center (*n* = 1, 4%) (29). The majority took place in rural settings (*n* = 16, 64%) (28, 30, 31, 34, 35, 38, 39, 41, 42, 44–48, 50, 52), although the specific site was unspecified in 4 (16%) (35, 40, 52, 53). The duration of training was highly variable, ranging from 1 day to several years. Ten (40%) programs lasted a week or less (27, 29, 34, 35, 37, 40, 48, 49, 51, 53), with nearly all completed within 3 months (*n* = 22, 88%) (27, 29–31, 34–40, 43–53). Only 3 (12%) programs lasted longer than 6 months (28, 41, 42), with 2 (8%) of these lasting over a year (28, 42).

### Content of Training Curricula

All of the studies described initiatives that included training in ultrasound to determine gestational age, with the majority of programs (*n* = 18, 72%) focused solely on obstetric ultrasound (27, 28, 30, 31, 35, 36, 39–43, 45–47, 49, 51–53). Others (*n* = 7, 28%) were more diverse (29, 34, 37, 38, 44, 48, 50), including scanning for cervical length (*n* = 3, 12%) (37, 50, 55) and gynecological conditions such as fibroids (*n* = 2, 8%) (29, 38). A small number of studies (*n* = 3, 12%) reported multi-system training, including ultrasound to detect abnormalities in the renal and hepatobiliary systems (34, 48, 50). Even within the 18 studies focused exclusively on obstetric indications, there was still relative heterogeneity in the range of topics covered. A small proportion taught fetal biometry alone (*n* = 6, 33%) (31, 35, 43, 45, 49, 53), with one teaching practitioners to perform “sweeps” of the maternal abdomen to permit measurements to be performed by trained staff working remotely (51). The remainder (*n* = 11, 61%) covered a wider range of obstetric topics including placental site and amniotic fluid index (27, 28, 30, 36, 39–42, 46, 47, 52). Detailed information pertaining to the content of the different curricula is provided in Table 1.

**Table 1:** Specific topics covered within each training programme (presented alphabetically by first author).

			**Content of Curricula**										
**References**	**Duration of training**	**Previous experience of USS**	**Machine safety and set up**	**Early pregnancy complications**	**Identification of multiple pregnancy**	**Fetal presentation**	**Amniotic fluid index**	**Placental site**	**Fetal biometry**	**Fetal anomalies**	**Cervical length**	**Gynecology**	**Other systems**
Adler et al. (34)	4 days					X			X				X
Ahmadzia et al. (27)	1 day	X	X			X	X	X	X				
AMANHI Group (35)	4 days	X							X				
Baj et al. (36)	8 weeks		X	X	X	X	X	X	X	X			
Bentley et al. (37)	1 week		X	X	X	X		X	X		X		
Boamah et al. (28)	2 years				X			X	X				
Enabudoso and Adams (29)	5 days	X	X						X	X		X	
Greenwold et al. (38)	8 weeks		X	X	X	X		X	X			X	
Kawooya et al. (39)	3 months		X		X	X	X	X	X	X			
Kinnevey et al. (30)	6 weeks		X						X	X			
Kim et al. (40)	2 days				X		X		X	X			
Kimberly et al. (41)	6 months		X		X	X		X	X				
Lee et al. (42)	3 years		X		X	X	X	X	X				
Mashamba (52)	12 weeks								X	X			
Millar et al. (43)	2 weeks	X							X				
Nathan et al. (44)	2 weeks		X		X	X	X	X	X	X	X		
Neufeld et al. (45)	6 weeks		X						X				
Rijken et al. (31)	3 months								X				
Sarris et al. (53)	3 days	X							X				
Shah et al. (50)	9 weeks		X	X		X		X	X		X		X
Shah et al. (46)	2 weeks		X		X	X	X	X	X				
Toscano et al. (51)	1 day		X										
Vinayak et al. (47)	4 weeks		X		X	X	X	X	X				
Wanjiku et al. (48)	1 day					X		X	X				X
Wylie et al. (49)	1 week								X				

### Components of Training Programs

Table 2 illustrates the individual components encompassing each of the training packages, highlighting a number of consistencies between the programs, especially with regard to training methodologies. Twenty-three programs made some reference to didactic teaching (27–31, 34, 35, 37–42, 44, 46, 47, 49–53), be this in person or online and nearly all (*n* = 22, 88%) described the inclusion of supervised “hands on” training (27–29, 31, 34, 35, 37–39, 41–53).

**Table 2:** Specific components of training programmes (presented alphabetically by first author).

	**Components of training programme**													
	**Logistics**		**Teaching methodology**				**Assessment**					**Follow up**		
**References**	**Training delivered in clinical setting**	**Training delivered by local practitioners**	**Pre course learning**	**e- learning**	**Didactic training**	**Supervised hands on training**	**Knowledge assessment**	**Practical assessment**	**Assessment of trainee confidence**	**Matched pre/post course testing**	**Evaluation of training**	**Supervision or mentorship**	**Remote image review**	**Subsequent retesting**
Adler et al. (34)	X				X	X								
Ahmadzia et al. (27)	X				X	X	X		X	X	X			
AMANHI Group (35)					X	X	X	X		X		X	X	
Baj et al. (36)	ECUREI	X		X							X			
Bentley et al. (37)	X				X	X	X	X	X	X				X
Boamah et al. (28)	X				X	X						X	X	
Enabudoso and Adams (29)		X	X		X	X			X		X			
Greenwold et al. (38)	X				X	X						X	X	
Kawooya et al. (39)	ECUREI	X			X	X						X		
Kinnevey et al. (30)	ECUREI	X			X				X					
Kim et al. (40)		X			X		X		X	X	X			
Kimberly et al. (41)	X				X	X		X				X		
Lee et al. (42)		X		X	X	X	X	X				X	X	
Mashamba (52)		X			X	X						X		
Millar et al. (43)	X	X				X		X				X	X	
Nathan et al. (44)	X	X			X	X	X	X				X	X	X
Neufeld et al. (45)		X				X		X				X		
Rijken et al. (31)	X	X			X	X	X	X				X	X	
Sarris et al. (53)		X			X	X		X				X	X	
Shah et al. (50)	X				X	X						X	X	
Shah et al. (46)	X	X			X	X		X	X	X		X	X	
Toscano et al. (51)	X				X	X							X	
Vinayak et al. (47)	X	X	X	X	X	X	X	X				X	X	
Wanjiku et al. (48)	X		X	X		X	X	X				X		X
Wylie et al. (49)	X				X	X						X	X	

### Assessments

Despite the majority of studies describing improvements in trainees' knowledge and skill after training, not all provided data to support this, with 11 (44%) studies failing to carry out any trainee evaluation (28–30, 34, 36, 38, 39, 49–52). The remainder (*n* = 14, 56%) (27, 31, 35, 37, 40–48, 53) undertook some form of assessment ranging from written tests (*n* = 9, 64%) (27, 31, 35, 37, 40, 42, 44, 47, 48), to supervised practical exams (*n* = 12, 86%) (31, 35, 37, 41–48, 53) or a combination of both (*n* = 7, 50%) (31, 35, 37, 42, 44, 47, 48). Of the programs which undertook practical assessments, 5 (36%) did so in the format of Observed Structured Clinical Examinations (OSCEs) (37, 41, 42, 46, 48).

### Training Delivered in Line With Recommended Standards

Ten (40%) of the studies reported training that incorporated both didactic and “hands on” components, as well as some form of practical assessment (31, 35, 37, 41, 42, 46–48, 53, 55), however in only 1 (28) did trainees undertake the minimum number of supervised scans recommended by the WHO.

### Success of Training

Despite making efforts to assess the trainees, only five studies (36%) specified a pass mark (42–44, 46, 53). Of the 103 trainees assessed within these five studies, 99 (96%) passed. Despite 18 studies providing follow up (28, 31, 35, 38, 41–53, 56), only 3 (12%) arranged for repeat assessments to explore the retention of knowledge and skills (37, 48, 55). Of these only one specified a pass mark (44). Of the 40 trainees who were reassessed within this programme, all retained their competency. Further detail pertaining to programme assessment and follow up is shown in Supplementary Table 3.

### Barriers and Facilitators

Alongside the evaluation of the training itself, four studies (16%) also explored what factors influenced the delivery of the training, providing detailed descriptions of implementation and reporting outcomes in the context of recognized frameworks (40, 43, 46, 53).

Time for faculty to deliver and practitioners to attend training was cited as a significant barrier, with staff reporting concerns about competing priorities both in terms of attempting to incorporate ultrasound into routine services and in the provision of ongoing supervision and support (40, 43, 46).

The attitude and perception of individuals undergoing training was identified as a key factor, with those who were enthusiastic and open-minded about the provision of ultrasound acting as strong facilitators of the programs (39, 52). Conversely, staff who were resistant to change or resentful of being asked to undertake extra work led to barriers for implementation (39, 42, 45). Empowering trainees to take ownership of the programs, especially with regards to the organization and scheduling of the service, was reported as an important approach in mitigating some of these issues (42, 45, 52), as did the provision of regular feedback (42, 52). Ensuring training was delivered in partnership with, and supported by local teams was also cited as important in ensuring longevity of programs (42, 52), helping to facilitate regular access to consumables and adequate referral systems for when concerns were raised.

Finally, the cost of training was reported as an important barrier to the sustainable delivery of training and wider implementation of ultrasound (42).

## Discussion

### Key Findings

Despite similarities in pedagogical approach, we identified substantial heterogeneity in the content and duration of the programs and the way in which they assessed participants. Less than half of the initiatives adhered to the ISUOG recommendation that training incorporate both didactic and “hands on” components, as well as practical assessment, and in only one programme (28) did trainees perform the number of supervised scans recommended by the WHO (16). Within the programs that referenced specific requirements to pass (*n* = 5, 20%) (42–44, 46, 53), 96% did so, however the remaining 80% (*n* = 20) of programs did not report such outcomes, making it near impossible to evaluate “success.” Overall, this review highlights an inconsistent approach to the delivery and reporting of training in ultrasound to determine gestational age, at odds with international recommendations.

### Ensuring Quality

#### Training Methodology—“Hands on” Teaching and Assessment

Given that ultrasound examinations are an important component of obstetric decision making, it is of paramount importance that they are of sufficient quality. As the accuracy of ultrasound is primarily dependent on the skill of the operator (57), adequate training is essential. While there is no universally agreed or standardized approach to training, nor a specific definition as to what constitutes competency to perform independent ultrasound examinations, there are some recommendations which seek to ensure that practitioners are appropriately trained and have demonstrated adequate proficiency prior to performing scans independently (15, 16, 57).

While only 10 programs (40%) incorporated all three of the components recommended by the ISUOG (31, 35, 37, 41, 42, 46–48, 53, 55), the combination of didactic and “hands on” training was adopted by 22 (88%) (27–29, 31, 34, 35, 37–39, 41–53) meaning it was predominantly the lack of trainee assessment that resulted in programs to failing to meet the required standards. Indeed, even amongst those who did perform assessments, the absence of criteria to “pass” makes it impossible to know whether training had been successful and if trainees were truly competent to perform scans independently. As such, it appears that the majority of practitioners trained by these initiatives have not met either ISUOG or WHO standards, a finding in keeping with previous work undertaken in 2012 by Lagrone et al. (58). Although undertaking 200 supervised ultrasound scans may not necessarily be achievable in many LMIC healthcare systems, delivering training that involves didactic and “hands on” components, as well as robust assessments should be. We believe this should be an important focus to improve the quality of future initiatives.

#### Ongoing Mentorship and Quality Assurance

Another key factor in ensuring both the quality and longevity of programs is the support provided to trainees at the end of the training period, helping to build confidence and ensure examinations continue to be of an appropriate standard (43, 46). Recent advances in tele-radiology have played a huge part in enabling this, presenting a meaningful solution to the ways in which programs can overcome the challenge of providing ongoing supervision in remote geographical locations or when faculty are scarce. Thirteen of the programs included in this review describe the transfer of ultrasound images (28, 31, 35, 38, 42–44, 46, 47, 49, 50, 53) for remote review, with feedback provided *via* the same platform. This approach appears to help reinforce positive practice and address areas for improvement where necessary. Although dependent on adequate internet coverage, the majority of programs employing these techniques reported successful implementation. Indeed, with access to smartphones ever expanding, this relatively simple approach may provide a cost effective solution to improving support and mentorship for all manner of training programs.

### Sustainability/Embedding in Pre-existing Services

The literature surrounding the delivery of successful and sustainable programs, suggests that a thorough consideration of how training can be supported, delivered and integrated within the resource constraints of pre-existing healthcare systems is essential (14, 59). The involvement of local practitioners and key stakeholders from the outset is important in ensuring programs are able to correctly prioritize context-specific training needs and focus only on what is necessary for the local population (60, 61), a concept supported by the qualitative findings of Shah et al. (46). Likewise by empowering and assisting local teams to develop training, programs are also able to ensure the design and delivery of materials are socially and culturally relevant and communities are adequately engaged in the expansion of new services (11, 14, 62). Although local teams were involved in the design of 10 (29–31, 36, 39, 40, 42, 44, 52, 53) and the delivery of 14 of the studies (29–31, 36, 39, 40, 42–47, 52, 53) the majority were partnered with overseas institutions (30, 31, 39, 42–47, 53), highlighting the complexity of establishing truly native initiatives. Central to this is the ability of groups to access adequate financial support, often granted preferentially to teams partnered with institutions from high income settings (63). Access to sufficient and sustainable financing programs is essential, not only to establish training at an individual level, but to upscale, embed and maintain the provision of ultrasound services thereafter (64, 65). Although there are numerous benefits to collaboration, these alliances are not without challenges and care must be taken to ensure they are balanced and that oversight and ownership remains with the LMIC partner (61, 66–68).

### Strengths and Limitations

The substantial discrepancy in the depth and quality of information provided by individual studies may have risked the misinterpretation of some findings, and the inability to contact authors for verification led to the exclusion of nine programs which may have been relevant. Furthermore, there were great disparities in the way studies reported findings, again limiting direct comparisons. In only representing programs which have been reported, this review is subject to a degree of publication bias, exacerbated by the fact most papers were written in English by authors from British or American institutions. The fact that most studies describe collaborations with overseas institutions further alludes to the potential omission of indigenous programs, which appear underrepresented in the literature. It is also likely that much training is delivered *ad hoc* in an apprenticeship-type model, which was not captured in this review. By predominantly summarizing training delivered within the context of research projects, it is also possible that results have been confounded by the additional allocation of resources afforded by study activities and may not be truly representative of the “real world” context.

That said, our review aimed to be as inclusive as possible and as such, incorporated descriptions of training from a wide variety of sources and settings. Whilst the heterogeneity of our results made direct comparisons challenging, this is the first systematic review to focus specifically on the provision of training in ultrasound to determine gestational age. Our findings therefore, have enabled us to provide valuable insight into what should constitute best practice in the development and reporting of training programs and indeed what may be required to upscale them.

### Recommendations

Having collated our results and found significant disparity in the quality of data, we have generated key recommendations for the reporting of training in basic obstetric ultrasound, presented in the context of the RE-AIM (Reach, Effectiveness, Adoption, Implementation and Maintenance) framework (Table 3). RE-AIM is an implementation tool which has been used extensively in both high- and low- income settings for the evaluation of skills training (40, 69–73), helping to facilitate the translation of research into practice.

**Table 3:** Recommendations for the design and delivery of ultrasound training programmes presenting within the RE-AIM framework.

**Recommendation**	**Description of recommendation**
Reach	*Providers* • Who developed the training? • What are the qualifications/experience of those providing the training? • Which local stakeholders were involved in its organization and delivery?
	*Participants* • Who participated in the training (demographic characteristics)? • How were they recruited? • Which individuals were included or excluded in the training? Why? • What proportion of eligible participants received the training? • What prior experience did they have? • What are their qualifications? • Were they given any incentive to participate?
Effectiveness or efficacy	•How were participants assessed and by whom? • What was the pass mark? How was this determined? • Describe what follow up was undertaken • Were trainees reassessed? • If reassessed what was the retention rate of skills/knowledge? • Were there any quality assurance processes? • Did the participants receive any formal certification or accreditation? If so, who bestowed this?
Adoption	*Setting level* • Where was the training delivered? • Which sites were included or excluded in the intervention? Why? • Describe the characteristics of the participating sites • What site preparation was undertaken prior to the training?
	*Individual level* • What proportion of those invited to participate completed the training? • Describe individuals' feedback on their experience of participating in the training
Implementation	*Content and setting* • Provide a brief description of the purpose of the training • Describe the learning objectives and how the training priorities were established • Describe the specific training materials provided to both the faculty and the participants and how these were developed
	*Education methodology* • How was the training delivered? (lectures, small group sessions, “hands on” practice, level of direct supervision, etc.) • What was the ratio of trainers to trainees? • Indicate how many ultrasound examinations were performed by each trainee and what proportion of these were directly supervised
	*Fidelity* • What percent of training delivery adhered to the original protocol? • Did the training require any adaptation or modification? If so, describe and explain the rationale for changes
	*Costs* • Who funded the training? • What was the final cost of the training?
Maintenance	•What consideration was given to factors affecting the delivery of the training? • What consideration was given to the ongoing provision of ultrasound and its integration into pre-existing services? • Were these studied formally?
	*Individual level* • What is the percentage of skills/knowledge retention amongst participants at or beyond 6 months from original ultrasound training?
	*Setting level* • Is the program ongoing 6 months post formal study funding? • Has ultrasound training/provision been adapted into the local setting over time?

## Conclusion

There is substantial heterogeneity in the current approach to training practitioners to determine gestational age using ultrasound in LMICs, with many programs failing to meet international recommendations for the delivery of safe and sustainable training programs. Our review highlights the need for a more consistent approach and has identified key areas we believe should be the focus of future initiatives to deliver high quality training in basic obstetric ultrasound. With an increasing focus on the importance of improving estimates of gestational age in LMICs, we believe this review will be of interest to those seeking to develop and expand the provision of basic obstetric ultrasound in LMICs.

## Data Availability Statement

The original contributions presented in the study are included in the article/Supplementary Material, further inquiries can be directed to the corresponding author/s.

## Author Contributions

AV conceived and developed the study with support from JN, SS, and RR, performed the searches, with both AV and IO screening the results for inclusion, and drafted the manuscript with editorial input from IO, JN, SS, and RR. Data was extracted by AV and verified by IO, with both AV and IO grading the quality of the included studies. Overall support and oversight was provided by JN, SS, and RR. All authors contributed to the article and approved the submitted version.

## Funding

This work was undertaken with support from the National Institute for Health Research (NIHR) (GHR Project: 17/63/08 DIPLOMATIC collaboration) using UK aid from the UK Government to support global health research. In addition, AV received funding from The McKern Fellowship, SS from The Wellcome Trust, and RR from the British Heart Foundation.

## Author Disclaimer

The views expressed in this publication are those of the authors and not necessarily those of the NIHR or the Department of Health and Social Care.

## Conflict of Interest

The authors declare that the research was conducted in the absence of any commercial or financial relationships that could be construed as a potential conflict of interest.

## Publisher's Note

All claims expressed in this article are solely those of the authors and do not necessarily represent those of their affiliated organizations, or those of the publisher, the editors and the reviewers. Any product that may be evaluated in this article, or claim that may be made by its manufacturer, is not guaranteed or endorsed by the publisher.
