# Supplementary material for: Training in Ultrasound to Determine Gestational Age in Low- and Middle- Income Countries: A Systematic Review
Source: Front Glob Womens Health. 2022 Mar 18;3:854198. doi: 10.3389/fgwh.2022.854198 (PMC8971706; doi:10.3389/fgwh.2022.854198)
Supplement: Supplementary file 1 [file Table_1.DOCX]

**Supplementary Table 1. Details of search strategy**

| **Database** | **Search terms used and limits applied** | **Date of last search** | **Number of records returned** |
| --- | --- | --- | --- |
| EMBASE | low income country*.mp. OR income*.mp. or Income/ OR family income*.mp. OR household income*.mp. OR income group*.mp. OR middle income group*.mp. OR middle income country*.mp. OR developing country*.mp. or developing countries/ OR rural*.mp. OR rural area*.mp. OR rural health*.mp. or rural health/ OR rural health services*.mp. or rural health services/ OR rural population*.mp. or rural population/ OR refugee*.mp. or refugees/ OR migrant*.mp. or “transients and migrants”/ OR low resource*.mp. OR deprived*.mp. OR poverty*.mp. or poverty/ OR under resourced*.mp. OR Afghanistan*.mp. or afghanistan/ OR Albania*.mp. or albania/ OR Algeria*.mp. or algeria/ OR American samoa*.mp. or american samoa/ OR Angola*.mp. or angola/ OR Argentina*.mp. or argentina/ OR Armenia*.mp. or armenia/ OR Azerbaijan*.mp. or azerbaijan/ OR Bangladesh*.mp. or bangladesh OR Belarus*.mp. or belarus OR Belize*.mp. or belize/ OR Benin*.mp. or benin/ OR Bhutan*.mp. or bhutan/ OR Bolivia*.mp. or bolivia/ OR “Bosnia and Herzegovina”/ or bosnia*.mp. OR Botswana*.mp. or botswana/ OR Brazil*.mp. or brazil/ OR Bulgaria*.mp. or bulgaria/ OR Bukina Faso*.mp. OR Burundi*.mp. or burundi/ OR Cabo verde*.mp. OR Cambodia*.mp. or cambodia/ OR Cameroon*.mp. or cameroon/ OR Central african republic*.mp. or central african republic/ OR Chad*.mp. or chad/ OR China*.mp. or china/ OR Colombia*.mp. or colombia/ OR Comoros*.mp. or comoros/ OR Congo*.mp. or congo/ OR Democratic republic congo*.mp. or democratic republic congo/ OR costa rica*.mp. or costa rica/ OR cote d'ivoire*.mp. or cote d'ivoire/ OR cuba*.mp. or cuba/ OR africa*.mp. or africa/ OR (africa adj3 sahara).mp. OR north africa*.mp. or northern africa/ OR south africa*.mp. or south africa/ OR Central africa*.mp. OR Djibouti*.mp. or djibouti/ OR Dominica*.mp. or dominica/ OR Dominican republic*.mp. or dominican republic/ OR ecuador*.mp. or ecuador/ OR egypt*.mp. or egypt/ OR El salvador*.mp. or el salvador/ OR Equatorial Guinea*.mp. or equatorial guinea/ OREritrea*.mp. or eritrea/ OR Eswatini*.mp. OR Ethiopia*.mp. or ethiopia/ OR swaziland*.mp. or swaziland/ OR fiji*.mp or fiji/ OR gabon*.mp. or gabon/ OR gambia*.mp. or gambia/ OR georgia*.mp. or georgia/ OR ghana*.mp. or ghana/ OR grenada*.mp. or grenada/ OR guatemala*.mp. or guatemala/ OR new guinea*.mp. or new guinea/ OR Guinea-Bissau*.mp. or guinea-bissau/ OR guyana*.mp. or guyana/ OR Haiti*.mp. or haiti/ OR Honduras*.mp. or honduras/ OR India*.mp. or india/ OR Indonesia*.mp. or indonesia/ OR Iran*.mp. or iran/ OR Islamic Republic*.mp. OR iraq*.mp. or Iraq/ OR jamaica*.mp. or jamaica/ OR jordan*.mp. or jordan/ OR Kazakhstan*.mp. or kazakhstan/ OR kenya*.mp. or kenya/ OR kiribati*.mp. or kiribati/ OR korea*.mp. or korea/ OR Kosovo*.mp. or kosovo/ OR Kyrgyz republic*.mp. or kyrgyz republic/ OR (republic adj2 lao).mp. OR Lebanon*.mp. or lebanon/ OR libya*.mp. or libya/ OR Madagascar*.mp. or madagascar/ OR malawi*.mp. or malawi/ OR malaysia*.mp. or malaysia/ OR maldives*.mp. or maldives/ OR Mali/ OR marshall islands*.mp. or marshall islands/ OR mauritania*.mp or mauritania/ OR mauritius*.mp. or mauritius/ OR carribean islands*.mp. or carribean islands/ OR indian ocean*.mp. or indian ocean/ OR (federated adj3 micronesia).mp. OR Mexico*.mp. or mexico/ OR new mexico*.mp. or new mexico/ OR (state adj2 mexico).mp. OR (gulf adj2 mexico).mp. OR Mexico city*.mp. or mexico city/ OR Moldova*.mp. or moldova/ OR Mongolia*.mp. or mongolia/ OR inner mongolia*.mp. or inner mongolia/ OR Montenegro*.mp. or montenegro/ OR Morocco*.mp. or morocco/ OR Mozambique*.mp. or mozambique/ OR myanmar*.mp. or myanmar/ OR Namibia*.mp. or namibia/ OR Nauru*.mp. or nauru/ OR Nepal*.mp. or nepal/ OR Nicaragua*.mp. or nicaragua/ OR Niger*.mp. or niger/ OR Nigeria*.mp. or nigeria/ OR North Macedonia*.mp. or macedonia republic/ OR Pakistan*.mp. or pakistan/ OR Papua New Guinea*.mp. or papua new guinea/ OR Paraguay*.mp. or paraguay/ OR (Peru* or peru).mp. OR Philippines*.mp. or phillipines/ OR Romania*.mp. or romania/ OR Rwanda*.mp. or rwanda/ OR Russian Federation*.mp. or russian federation/ OR USSR*.mp. or USSR/ OR Samoa*.mp. or samoa/ OR São Tomé*.mp. OR Senegal*.mp. or senegal/ OR Serbia*.mp or serbia/ OR Sierra Leone*.mp. or sierra leona/ OR Solomon Islands*.mp. or solomon islands/ OR Somalia*.mp or somalia/ OR South Sudan*.mp. or south sudan OR Sri Lanka*.mp or sri lanka OR St. Lucia*.mp. OR (vincent adj2 grenadines).mp. OR sudan*.mp. or sudan/ OR Suriname*.mp. or suriname/ OR syrian Arab Republic*.mp. or syrian arab republic/ OR Tajikistan*.mp. or tajikistan/ OR Tanzania*.mp. or tanzania/ OR Thailand*.mp. or thailand/ OR Timor-Leste*.mp. or timor leste/ OR Togo*.mp. or togo/ OR Tonga*.mp. Or tonga/ OR Tunisia*.mp. or tunisia/ OR Turkey*.mp. or turkey/ OR Turkmenistan*.mp. or turkmenistan/ OR tuvalu*.mp. or tuvalu/ OR Uganda*.mp. or uganda/ OR Ukraine*.mp. or ukraine/ OR Uzbekistan*.mp. or uzbekistan/ OR Vanuatu*.mp. or vanuatu/ OR Venezuela*.mp. or venezuela/ OR Vietnam*.mp. or vietnam/ OR (west bank and gaza*).mp. OR yemen*.mp. or yemen/ OR Zambia*.mp. or zambia/ OR Zimbabwe*.mp. or zimbabwe/ OR lesotho*.mp. Or lesotho/ OR (Liberia* or Liberia).mp.  AND  gestation*.mp. OR gestation period*.mp. OR gestational age*.mp or gestational age/ OR pregnancy*.mp. OR pregnancy/ OR (estimated adj3 delivery).mp. OR estimated due date*.mp. OR due date* OR confinement*.mp. OR conceptional age*.mp. OR prenatal*.mp.  AND  fetal*.mp. OR obstetric*.mp.  AND  ultrasound*.mp OR scan*.mp OR scanning*.mp. OR sonogram*.mp. OR ultrasonogrphy*.mp or ultrasonography/ or ultrasonography, prenatal.mp. OR sonography*.mp OR portable ultrasound scanner*.mp OR ultrasound scanner*.mp. OR ultrasonographic*.mp.  AND  training*.mp. OR curriculum*.mp. or curriculum/ OR education*.mp. or education/ OR teaching*.mp. or teaching/ OR (learning* or learning).mp. OR course*.mp. OR qualification*.mp. OR programme development*.mp. or program development/ OR supervision*.mp. OR mentoring*.mp. OR simulation*.mp. or simulation training/ | 12/7/21 | 562 |
| AMED | Search terms as per EMBASE | 12/7/21 | 0 |
| MEDLINE | Search terms as per EMBASE | 12/7/21 | 288 |
| CINAHL | low and middle income countries/ or low income country OR income/ or income OR rural health or rural health centers/ or rural areas/ or rural health services/ or hospitals, rural/ or rural health personnel/ or rural population/ OR low resource settings OR deprived OR under resourced OR developing countries/ or developing countries OR refugee or refugees/ OR poverty/ or poverty or poverty areas/ OR Afghanistan or afghanistan/ OR Albania or albania/ OR American samoa or american samoa/ OR Algeria or algeria/ OR Angola or angola/ OR Argentina or argentina/ OR Armenia or armenia/ OR Azerbaijan or azerbaijan/ OR Bangladesh or bangladesh/ OR Belarus or byelarus/ OR Belize OR Benin OR Bhutan or bhutan/ OR Bolivia OR Bosnia OR Botswana OR Brazil or brazil/ OR Bulgaria OR Bukina Faso/ or bukina faso OR Burundi/ or Burundi OR Cabo verde or cape verde/ OR Cambodia OR Cameroon or cameroon/ OR Central african republic or central african republic/ OR Chad OR China or china/ OR Colombia/ OR Comoros OR Congo or congo/ or demographic republic of congo/ OR costa rica or costa rica/ OR cote d'ivoire or cote d'ivoire/ OR  cuba OR africa or africa/ or africa south of the sahara/ OR north Africa OR south africa or south africa/ or africa, southern OR africa, western/ OR Central africa or africa, central/ OR Djibouti or djibouti/ OR Dominica or dominica/ OR Dominican republic or dominican republic/ OR Ecuador/ OR Egypt/ or eygpt OR El Salvador OR Equatorial Guinea or equatorial guinea/ OR Eritrea or ertrea/ OR Eswatini OR Ethiopia OR swaziland OR menalasia/ OR gabon OR gambia/ or gambia OR georgia or georgia/ or georgia, republic/ OR ghana or ghana/ OR grenada OR Guatemala OR new guinea or new guinea/ OR Guinea-Bissau or guinea bissau/ OR Guyana OR Haiti OR Honduras OR India or india/ OR Indonesia or indonesia/ OR Iran OR Islamic Republic OR iraq OR Jamaica OR jordan or jordan/ OR Kazakhstan OR kenya/ OR Kiribati OR Korea OR Kosovo or yugoslavia/ OR Kyrgyz republic OR republic adj2 lao or laos/ OR Lebanon OR Libya OR Madagascar/ OR malawi/ OR malaysia or malaysia/ OR Maldives OR marshall islands OR Mauritania/ OR mauritius or indian ocean islands/ OR carribean islands OR federated adj3 micronesia or micronesia/ OR Mexico or mexico/ or new mexico/ OR Moldova OR Mongolia OR inner Mongolia OR Montenegro OR Morocco/ OR Mozambique/ OR myanmar/ OR Namibia/ OR Nauru OR Nepal/ OR Nicaragua/ OR Niger/ OR Nigeria/ OR North Macedonia OR Pakistan/ OR Papua New Guinea or papua new guinea/ OR Peru OR Philippines OR Romania OR Rwanda/ OR Russian Federation OR USSR/ or USSR OR Samoa OR Sao Tome OR Senegal OR Serbia OR Sierra Leone OR Solomon Islands OR Somalia/ OR South Sudan OR Sri Lanka/ OR St Lucia OR vincent adj2 grenadines OR Suriname/ OR syrian Arab Republic OR Tajikistan OR Tanzania/ OR Thailand/ OR Timor-Leste or east timor/ OR Togo/ OR polynesia/ OR Tunisia OR Turkey/ OR Turkmenistan/ OR Tuvalu OR Uganda/ OR Ukraine OR Uzbekistan/ OR Vanuatu OR Venezuela/ OR Vietnam OR west bank and gaza OR yemen OR Zambia/ OR Zimbabwe OR lesotho/ OR Liberia/  AND  Gestation OR estimated adj3 delivery OR estimated due date OR due date OR confinement OR conceptional age OR prenatal OR pregnancy/  AND  Fetal OR obstetric or obstetric care/  AND  ultrasound OR scan OR scanning OR sonogram OR ultrasonography OR sonography OR portable ultrasound scanner OR ultrasound scanner OR  ultrasonographic  AND  Training OR curriculum or curriculum/ or course content/ OR education OR  teaching OR learning/ OR course OR qualification OR programme development OR supervision or supervisors and supervision/ OR  mentoring or mentorship/ OR simulation | 12/7/21 | 60 |
| AIM | gestation OR gestation period OR gestational age OR pregnancy OR estimated due date OR due date OR confinement OR conceptional age OR prenatal  AND  fetal or obstetric  AND  ultrasound OR scan OR scanning OR sonogram OR ultrasonography OR sonography OR portable ultrasound scanner OR ultrasound scanner OR ultrasonographic  AND  training OR curriculum OR education OR teaching OR learning OR course OR qualification OR programme development OR supervision OR mentoring OR simulation | 12/7/21 | 0 |
| Global Index Medicus | Search terms as per AIM | 12/7/21 | 90 |
| Cochrane | low income country OR lowest income group IR income OR family income OR gross national income OR household income OR income group OR middle income group OR middle income country OR developing country OR rural OR rural area OR rural health OR rural healthcare OR rural population OR refugee OR migrant OR low resource OR deprived OR poverty OR under resourced  AND  gestation OR gestation period OR gestational age OR pregnancy OR estimated due date OR due date OR confinement OR conceptional age OR prenatal  AND  fetal or obstetric  AND  ultrasound OR scan OR scanning OR sonogram OR ultrasonography OR sonography OR portable ultrasound scanner OR ultrasound scanner OR ultrasonographic  AND  training OR curriculum OR education OR teaching OR learning OR course OR qualification OR programme development OR supervision OR mentoring OR simulation | 12/7/21 | 48 |
| Web of Science | All=(low income country OR lowest income group OR income OR family income OR gross national income OR household income OR income group OR middle income group OR middle income country OR developing country OR rural OR rural area OR rural health OR rural healthcare OR rural population OR refugee OR migrant OR low resource OR deprived OR poverty OR under resourced)  AND  All=(gestation OR gestation period OR gestational age OR pregnancy OR estimated due date OR due date OR confinement OR conceptional age OR prenatal)  AND  All=(fetal or obstetric)  AND  All=(ultrasound OR scan OR scanning OR sonogram OR ultrasonography OR sonography OR portable ultrasound scanner OR ultrasound scanner OR ultrasonographic)  AND  All=(training OR curriculum OR education OR teaching OR learning OR course OR qualification OR programme development OR supervision OR mentoring OR simulation) | 12/7/21 | 214 |
