# Supplementary material for: Training in Ultrasound to Determine Gestational Age in Low- and Middle- Income Countries: A Systematic Review
Source: Front Glob Womens Health. 2022 Mar 18;3:854198. doi: 10.3389/fgwh.2022.854198 (PMC8971706; doi:10.3389/fgwh.2022.854198)
Supplement: Supplementary file 2 [file Table_2.docx]

*Table 2. Characteristics of included studies (presented alphabetically by first author)*

| **Authors** | **Study Design** | **Journal and year** | **Location** | **Number and cadre of Trainees** | **Previous experience of ultrasound** | **Recruitment of Trainees** | **Funding** | **Strengths** | **Limitations** | **MMAT Score** |
| --- | --- | --- | --- | --- | --- | --- | --- | --- | --- | --- |
| Adler(35) | Observational study  *Non randomised* | Int J Emerg Med  2008 | Lugufu Refugee Camp  Tanzania | 10 trainees  Physicians and clinical officers | Unspecified | Recruitment method unspecified | None | Detailed description of overall programme.  Trainees must achieve a certain number of supervised scans.  Attempt made to undertake follow up. | No practical or written assessment of skill.  No mentoring.  Follow up only concerned the use of the machine not the quality of the scans performed. | 2 |
| Ahmadzia(28) | Prospective cohort study  *Non randomised* | African Health Sciences  2018 | Mulago National Referral Hospital, Kampala  Urban Uganda | 40 trainees  Medical students and junior/senior house officers | Some had previous training or experience of using ultrasound | Voluntary  No criteria specified for participation | None | Matched pre and post intervention testing with responses from 95% of trainees. | Time between tests not standardised – No differentiation made for those who had previous experience of using ultrasound.  No opportunity for trainees to perform ultrasound themselves.  No practical assessment of skill.  No mentoring or follow up. | 2 |
| AMANHI group(36) | Prospective cohort study  *Non randomised* | The Lancet  2020 | Sylhet, Bangladesh.  Karachi, Pakistan.  Pemba, Tanzania.  Rural/Peri Urban | 10 trainees  Sonographers | All had previous training or experience of using ultrasound | Recruitment method unspecified | Bill and Melinda Gates Foundation | Written and practical trainee assessment. Matched pre and post testing.  Trainees mentored with ongoing quality assessment and feedback relating to their images. | Limited description of programme. | 4 |
| Baj(37) | Survey  *Quantitative descriptive* | Ultrasound  2015 | Ernest Cook Ultrasound Research and Education Centre( ECUREI) Kampala  Urban Uganda | Number of trainees unspecified  High school graduates | None had previous training or experience of using ultrasound | Voluntary  Must be USS naïve | None | Undertaken in established ultrasound training centre.  Detailed description of design of programme with careful consideration given to the rural low resource context. | Training not provided to the intended participant group.  No opportunity for trainees to perform ultrasound themselves.  No practical or written assessment of skill.  No mentoring or follow up.  No reporting or analysis of results of training. | - |
| Bentley(38) | Prospective cohort study  *Non randomised* | J Ultrasound Med  2015 | Teaching Hospital Monrovia  Urban Liberia | 31 trainees  Midwives | Unspecified | Voluntary  Participation extended to all midwives at the site | Health Education and Relief Through Teaching | Matched pre and post intervention testing  Practical trainee assessment.  Attempt made to undertake follow up. | Limited description of programme.  Unclear if trainees had prior experience in ultrasound.  Incomplete data capture for all trainees – Only 55% underwent practical assessment. Only 45% followed up.  No mentoring. | 2 |
| Boamah(29) | Case report  *Quantitative descriptive* | JMIR Res Protoc  2014 | Kintampo North Municipal and South District Hospitals,  Brong Ahafo Region  Rural Ghana | 15 trainees  Midwives | None had previous training or experience of using ultrasound | Voluntary  Participation extended to all midwives at the sites | National Institute of Environmental Health Sciences.  Global Alliance for Clean Cookstoves  Thrasher Research Fund | Clear description of training programme.  Trainees provided with extensive practical experience.  Trainees mentored with ongoing quality assessment and feedback relating to their images. | Training took place in phases over 2 years – May not be generalisable to other low resource settings.  Images reviewed but no formal practical assessment.  No reporting or analysis of results of training. | - |
| Enabudoso(30) | Survey  *Quantitative descriptive* | Tropical Journal of Obstetrics and Gynaecology  2017 | Conference Hall, University of Benin Teaching Hospital  Urban Nigeria | 67 trainees  Obstetricians, radiologists, sonographers and private practitioners | Some had previous training or experience of using ultrasound | Voluntary  Participants had to pay to attend (Approx. £225) | None | Detailed description of content of programme. | No practical or written assessment of skill.  No mentoring or follow up.  No reporting or analysis of results of training. | 1 |
| Greenwold(39) | Prospective  cohort study  *Non randomised* | International Journal of Obstetrics and Gynaecology  2014 | Mandimba Health Clinic, Mandimba District  Rural Mozambique | 9 trainees  Nurses and clinical officers | None had previous training or experience of using ultrasound | Recruitment method unspecified | Medical Aid Films.  MaMA Mozambique.  Sonosite. | Refresher sessions provided 4 months after initial training.  Discussed barriers and facilitators to implementing ultrasound. | No practical or written assessment of skill.  Reported detection rates for specific findings were unverified by experienced sonographers. | 3 |
| Kawooya(40) | Before and after study  *Non randomised* | Ultrasound Quarterly  2015 | Six health centres in Mpigi District  Rural Uganda  Training at Ernest Cook Ultrasound Research and Education Centre (ECUREI) Kampala | 14 trainees  Midwives | Unspecified | Recruitment method unspecified | General Electric.  Midwives Antenatal Ultrasound Project.  STRIDES for family health/USAID-Uganda | Undertaken in established ultrasound training centre.  Some but not all scan images were reviewed for quality. | Limited description of programme.  No practical or written assessment of skill. | 2 |
| Kim(41) | Mixed Methods study | BMC Medical Education  2021 | Urban Nepal | 228 trainees  Radiologists, physicians, nurses, midwives and paramedics | Unspecified | Voluntary  No criteria specified for participation | JW LEE Centre for Global Medicine of Seoul National University College of Medicine | Matched pre and post intervention testing.  Formal review of implementation reporting on implementation outcomes. | Limited description of training programme.  Extent of previous ultrasound experience unclear.  No opportunity for trainees to perform ultrasound themselves.  No practical assessment of skill.  Only presents scores for approx. 50% of participants. | 2 |
| Kimberly(42) | Before and after study  *Non randomised* | Ultrasound in Med and Biol  2010 | Kapiri District Hospital, Mukonchi Rural Health Centre, Nkole Rural health Centre  Rural Zambia | 21 trainees  Midwives | None had previous training or experience of using ultrasound | Recruitment method unspecified | None  Sonosite donated the  machines | Practical assessment of skill.  Attempt made to undertake follow up.  Some but not all scan images were reviewed for quality.  Discussed barriers and facilitators to implementing ultrasound. | Uneven range of ultrasound exposure during training.  No specification of the requirements to pass the assessments.  Incomplete data capture for all trainees – Only 52% followed up.  No mentoring. | 2 |
| Kinnevey(31) | Survey  *Quantitative descriptive* | International Journal of MCH and AIDS  2016 | Health centres in 11 districts  Rural Uganda  Training at Ernest Cook Ultrasound Research and Education Centre( ECUREI) Kampala | 12 trainees  Midwives | None had previous training or experience of using ultrasound | Voluntary  No criteria specified for participation | Uganda Protestant Medical Bureau | Undertaken in established ultrasound training centre. | Limited description of training programme.  No practical or written assessment of skill.  No mentoring or follow up.  No reporting or analysis of results of training. | 2 |
| Lee(43) | Case report  *Quantitative descriptive* | Global Humanitarian Technology Conference  2015 | Nursing School Bwindi  Rural Uganda | Number of trainees unspecified  Nurses | None had previous training or experience of using ultrasound | Compulsory  All nursing students | Imaging the World | Detailed description of overall programme.  Practical and written assessment of trainee assessment with specific requirements to pass. | Limited reporting of results of training. | - |
| Mashamba(53) | Before and after study  *Non randomised* | Poster presentation  2018 | Gauteng Province  South Africa | 10 trainees  Advanced midwives | Unspecified | Trainees selected  No criteria specified for participation | Unspecified | Trainees mentored. | No description of training programme or assessment process.  Unclear if trainees had prior experience in ultrasound. | - |
| Millar(44) | Mixed methods study | JMIR Res Protoc  2018 | Jacaranda Health Maternity hospital, Nairobi  Peri-urban Kenya | 7 trainees  Sonographers and nurse midwives | All had previous training or experience of using ultrasound | Trainees selected  Must have previous experience of using ultrasound | Bill and Melinda Gates Foundation | Clear description of training programme.  Pre implementation site assessments.  Practical assessment of skill with specific requirements to pass.  Trainees mentored with ongoing quality assessment and feedback relating to their images.  Refresher sessions provided 6 months after initial training.  Formal review of implementation reporting on implementation outcomes. | Limited reporting of results of training. | 5 |
| Nathan(45) | Prospective  cohort study  *Non randomised* | Curr Probl Diagn Radiol  2017 | Chimaltenango, Guatemala  Lusaka, Zambia  Western Province, Kenya  Thatta, Pakistan  Equateur, DRC  Rural sites | 41 trainees  Physicians, nurse midwives and trained birth attendants | None had previous training or experience of using ultrasound | Trainees selected  No criteria specified for participation | Bill and Melinda Gates Foundation | Detailed description of overall programme.  Pre implementation site assessments.  Written and practical trainee assessment with specific requirements to pass.  Trainees mentored with ongoing quality assessment of their images.  Strong collaboration with local teams. | Limited information about how the ultrasound training was delivered at each of the sites.  Many of the scans were performed out with the intended gestational age range. | 4 |
| Neufeld(46) | Before and after study  *Non randomised* | Ultrasound Obstet Gynecol  2009 | Rural Bangladesh  Training at the Atomic Energy Commission Obstetric Hospital, Dhaka | 9 trainees  Paramedics | None had previous training or experience of using ultrasound | Recruitment method unspecified | United Nations Children’s Fund.  UK MRC.  Swedish International Development Cooperation Agency | Trainees mentored.  Robust analysis of trainee vs trainer measurements. | Limited description of training programme or assessment.  If trainee measurements were poor they were excluded from review | 3 |
| Rijken(32) | Before and after study  *Non randomised* | Ultrasound Obstet Gynecol  2009 | Shoklo Malaria Unit, Thai-Burmese Border  Rural | 4 trainees  Nurses and high school graduates | None had previous training or experience of using ultrasound | Trainees selected  By interview based on  motivation | None  University Medical Centre Utrecht donated the  Machines | Undertaken in established ultrasound training centre.  Practical assessment of skill.  Robust analysis of trainee vs trainer measurements. | Limited description of training programme and assessment. | 3 |
| Sarris(55) | Prospective  cohort study  *Non randomised* | BJOG  2013 | Pelotas, Brazil. Nagpur India, Parklands Suburb, Nairobi Kenya  Multiple Urban Sites | 9 lead sonographers – At least 1 from each participating site  Unspecified number of additional sonographers from participating sites – Trained by lead sonographer from their site | All had previous training or experience of using ultrasound | Voluntary  Must be motivated and experienced | Bill and Melinda Gates Foundation | Detailed description of programme and assessment.  Practical assessment of trainee assessment with specific requirements to pass.  Trainees mentored with ongoing quality assessment of their images.  Formal review of implementation reporting on implementation outcomes.  Strong collaboration with local teams. |  | 5 |
| Shah(51) | Before and after study  *Non randomised* | BMC International Health and Human Rights  2009 | Kirehe and Rwinkwavu District Hospitals, Eastern Province  Rural Rwanda | 15 trainees  Physicians | Unspecified | Voluntary  Participation extended to all physicians at the site | Sonosite donated the  machines | Clear description of training programme.  Ongoing quality assessment review of proportion of images. | No practical or written assessment of skill.  Not all images were reviewed and no feedback provided to trainees. | 1 |
| Shah(47) | Mixed methods study | PLOS One  2020 | Public District Hospital and Health Centres, Busoga Region  Urban and rural Uganda | 25 trainees  Nurse midwives and physicians | None had previous training or experience of using ultrasound | Voluntary  Must provide antenatal care at the site | Bill and Melinda Gates Foundation | Clear description of training programme and rationale for iterative changes.  Written and practical trainee assessment with specific requirements to pass.  Trainees mentored with ongoing quality assessment and feedback relating to their images. | Survey data only available for 68% of trainees. | 4 |
| Toscano(52) | Observational study  *Non randomised* | BMC Pregnancy and Childbirth  2021 | Health Centre, Lima  Urban Peru | 2 trainees  Nurse and care technician | None had previous training or experience of using ultrasound | Recruitment method unspecified | Innovate Peru | Detailed description of programme.  All images reviewed.  Thorough reporting of results of image review. | No practical or written assessment of skill.  No feedback provided to trainees. | 3 |
| Vinayak(48) | Prospective  cohort study  *Non randomised* | Ultrasound in Med and Biol  2017 | Health Centres  Rural Kenya  Training in Aga Khan University Hospital | 3 trainees  Midwives | None had previous training or experience of using ultrasound | Voluntary  Must have passed e-module | Philips Medical Systems | Detailed description of programme and rationale for iterative changes.  Practical and written assessment of trainee assessment.  Trainees mentored with ongoing quality assessment of their images. | No specification of the requirements to pass the assessments. | 3 |
| Wanjiku(49) | Prospective  cohort study  *Non randomised* | BMC Health Services Research  2018 | Health Centres  Rural Kenya | 33 trainees  Clinical officers, nurses and community workers | None had previous training or experience of using ultrasound | Voluntary  No criteria specified for participation | DAK foundation. Rotary Club of Greater Sydney | Practical and written assessment of trainee assessment with specific requirements to pass. | Limited information about how the ultrasound training was delivered at each of the sites.  The length of time between the end of training and assessment was inconsistent between trainees. | 3 |
| Wylie(50) | Prospective  cohort study  *Non randomised* | Malaria Journal  2013 | Ndirande Antenatal Care Clinic, Blantyre  Urban Malawi | 4 trainees  Clinicians and nurses  None had previous training/experience of using ultrasound | None had previous training or experience of using ultrasound | Recruitment method unspecified | Sonosite donated the  Machines | Trainees mentored with ongoing quality assessment of their images | Limited description of training programme.  No practical or written assessment of skill.  Limited reporting of results of training. | 2 |

*Table 3. Studies which undertook formal trainee assessment or follow up (presented alphabetically by first author)*

| **Author** | **Year** | **Total number of participating trainees and number who sat assessment** | **Methods of assessment and definition of competency** | **Proportion of trainees who passed assessment/achieved competency** | **Subsequent supervision or follow up** | **Proportion of trainees who maintained competency on any subsequent retesting** |
| --- | --- | --- | --- | --- | --- | --- |
| Ahmadzia(28) | 2018 | 40 trainees  All 40 underwent written assessment | Written assessment only  Matched pre/post survey assessing self-assessment of ultrasound proficiency and knowledge  No definition of competency and no specific requirements in order to ‘pass’ | No specific requirements to ‘pass’  Mean improvement of 5.8 points for post training scores | No follow up described | No subsequent retesting described |
| AMANHI Group(36) | 2020 | 10 trainees  All 10 underwent practical assessment  Unspecified number underwent written assessment | Written and practical assessment  Matched pre/post knowledge test  Fetal biometric measurements obtained by trainees compared with those of trainers  No definition of competency and no specific requirements in order to ‘pass’ | No specific requirements to ‘pass’  28% increase in post training knowledge scores | All study images were saved and transferred to the study supervisor for quality assessment undertaken against predefined criteria  Feedback was provided to the sonographers every quarter | No subsequent retesting described |
| Bentley(38) | 2015 | 31 trainees  All 31 underwent written assessment  17 trainees underwent practical assessment | Written and practical assessment  Matched pre/post knowledge test  OCSE assessment  No definition of competency and no specific requirements in order to ‘pass’ | No specific requirements to ‘pass’  Knowledge test mean score 90%  OSCE mean score 78% | All three components of assessment repeated 1 year later (Survey, knowledge test and OSCE)  Unclear if any subsequent communication between training team and trainees from the end of the course to the repeat testing | Written and practical assessment  repeated 1 year later  14/31 underwent knowledge test  Mean score 66%  8/31 underwent OSCE  Mean score 55% |
| Boamah(29) | 2014 | 15 trainees  Midwives | No formal assessment described | No specific requirements to ‘pass’  4 out of 15 midwives selected | All images transferred to the study supervisor for informal review every 2 weeks with feedback provided.  5% of all images were reviewed formally against predefined quality criteria | 3/4  1 sonographer was required to leave the programme due to a consistent inability to meet quality standards |
| Greenwold(54) | 2014 | 9 trainees  Nurses and clinical officers | No formal assessment described | No specific requirements to ‘pass’ | 10 month follow  Information on ultrasound findings collected and transferred to study team every week. Images also sent but unclear how these were assessed for quality | No subsequent retesting described |
| Kimberly(42) | 2010 | 21 trainees  17 trainees underwent practical assessment | Practical assessment only  OSCE assessment  No definition of competency and no specific requirements in order to ‘pass’ | No specific requirements to ‘pass’ | All images were saved during the independent scanning. 49% were reviewed  70% of BPD measurements were felt to be inaccurate and FL was not performed consistently enough to accurately determine gestational age  A follow up visit was undertaken 1 year after the training and midwives surveyed regarding their use of ultrasound following the training. 13/21 trainees responded | No subsequent retesting described |
| Kim(41) |  | 228 trainees  85 trainees underwent written assessment | Written assessment only  Matched pre/post knowledge test  No definition of competency and no specific requirements in order to ‘pass’ | No specific requirements to ‘pass’ | No follow up described | No subsequent retesting described |
| Lee(43) | 2015 | 22 nurses  All 22 underwent both written and practical assessment | Written and practical assessment  Trainees were required to achieve a score of 80% or above in both components to pass | 100% passed both components (22/22) | Longterm quality assurance programme  Trainees required to submit all images form first 100 independent scans for quality assessment and feedback.  No indication as to how these were assessed for quality.  Trainees are required to submit 2 images of each parameter per week thereafter for longterm quality assessment | No subsequent retesting described |
| Mashamba(53) | 2018 | 10 trainees  Advanced midwives | No formal assessment described | No specific requirements to ‘pass’ | 12 weeks of mentoring and assessment following initial training  No further information provided | No subsequent retesting described |
| Millar(44) | 2018 | 6 trainees  All 6 underwent practical assessment | Practical assessment only  Fetal biometric measurements obtained by trainees compared with those of trainers  Competency defined as the ability to perform three consecutive scans with ‘precise measurements and an accurate EDD’  No specific definition of what constituted ‘precise’ | 50% of trainees passed (3/6 trainees) | 12 months of follow up after initial training  Unspecified proportion of images transferred to study team for quality assessment undertaken against predefined criteria and feedback provided  Reinforcement training was provided by the study team 6 months after the initial training | No subsequent retesting described |
| Nathan(45) | 2017 | 41 trainees  All 41 underwent both written and practical assessment | Written and practical assessment  Trainees were required to achieve a score of 75% or above in both components to pass | 100% passed both components (41/41)  Written  95% on their 1^st^ attempt (39/41)  4% on their 2^nd^ attempt (2/41)  Practical  88% on their 1^st^ attempt (36/41)  12% on their 2^nd^ attempt (5/41) | 12 week post training follow up – Parent study pilot phase *(First Look Study)*  All images and their interpretations were transferred weekly to the study team for quality assessment against predefined criteria with feedback provided  Local trainers met with the trainees twice a week at the participating sites to troubleshoot and provide advice or ‘hands on’ assistance  Based on their submitted images some trainees were provided with targeted remedial assistance | Practical assessment was repeated 3 months later (40/41 trainees – one withdrew from study)  100% passed  Following this period 10% of all images recorded during the parent study were transferred for ongoing quality assessment and feedback |
| Neufeld(46) | 2009 | 9 trainees  All 9 underwent practical assessment | Practical assessment only  Fetal biometric measurements obtained by trainees compared with those of trainers  No definition of competency and no specific requirements in order to ‘pass’ | No specific requirements to ‘pass’  However 33% (3/9 trainees) were noted to have ‘larger errors’ and required additional training | No follow up described | No subsequent retesting described |
| Rijken(32) | 2009 | 4 trainees  All 4 underwent both written and practical assessment | Written and practical assessment  Post course knowledge test  Fetal biometric measurements obtained by trainees compared with those of trainers  A difference in measurements corresponding to a difference in gestational age +/- 7 days was considered acceptable, however there was no stipulation on how many times this must be achieved to pass | No specific requirements to ‘pass’ | Longterm quality assurance programme  All trainees required to submit 5 images of each parameter for review every 6 months and individual feedback is provided  No indication as to how these images are assessed for quality | No subsequent retesting described |
| Sarris(55) | 2011 | 9 trainees  All 9 underwent practical assessment | Practical assessment only  Fetal biometric measurements obtained by trainees compared with those of trainers and all images reviewed for quality against a set of criteria  Competency defined as   - The ability to perform three consecutive scans with measurements to within 1 standard deviation of the trainers - A score of 67% or more for each of their images | 100% passed both components (9/9) | Follow up throughout the duration of the parent study – *INTERGROWTH-21^st^*  Regular site visits by the study team to provide support, ensure ongoing functionality of ultrasound machines and to verify adherence to protocols  10% of all images recorded during the parent study were transferred for ongoing quality assessment and feedback against predefined criteria  If >10% of a sonographers images were scored poorly they would be recalled for re-training. If scores remained low their certification was withdrawn | No subsequent retesting described |
| Shah(51) | 2009 | 15 trainees  Physicians | No formal assessment described | No specific requirements to ‘pass’ | 10 week post training follow up  Unspecified proportion of images and a record of their interpretation transferred to the study team for review  No indication of how these were assessed for quality or if feedback was provided | No subsequent retesting described |
| Shah(47) | 2020 | 25 trainees  All 25 underwent practical assessment | Practical assessment only (must have undertaken 25 supervised scans prior to sitting assessment)  OSCE assessment  Trainees were required to achieve a score of 80% or above to pass | 96% trainees passed (24/25 trainees)  88% on their 1^st^ attempt (22/25)  8% on their 2^nd^ attempt (2/25) | 3 month post training follow up  For 8 weeks all images transferred to the study team for quality assessment undertaken by two trainers against predefined criteria  During this period the local lead sonographer also visited each site weekly to check the machines and to provide ‘hands on’ supervision for those who had been identified as requiring assistance based on the remote quality assessment of images  For 3 months after the training trainers communicated twice weekly with the trainees via WhatsApp to troubleshoot and offer advice on scans and to try to mitigate any issues | No subsequent retesting described |
| Vinayak(48) | 2017 | 3 trainees  All 3 underwent both written and practical assessment | Written and practical assessment  Pre course knowledge test  Post course knowledge test  OSCE assessment  Trainees were required to achieve a score of 100% to commence training  No definition of competency and otherwise no specific requirements in order to ‘pass’ | 100% passed the pre course knowledge test (3/3)  All 3 (100%) were reported to have passed the post course knowledge test and OSCE although no pass marks were specified. | Follow up throughout the duration of the study – Duration unspecified  All images and their interpretations transferred to the study team immediately after completion of the ultrasound scan. These were assessed immediately by two reviewers against predefined criteria who provided feedback in real time | No subsequent retesting described |
| Wanjiku(49) | 2018 | 33 trainees  All 33 underwent written assessment  20 trainees underwent practical assessment | Written and practical assessment  Matched pre/post knowledge test  OSCE assessment  Trainees were required to achieve a score of 90% or above  No definition of competency and no specific requirements in order to pass OSCE | 100% passed the pre course knowledge test (33/33)  27%% passed the post course knowledge test (9/33) | All assessments repeated 3-4 months later (Knowledge test and OSCE)  If they fail trainees are offered refresher teaching before further assessment 3-4 months later.  There does not appear to be a limit on the number of refresher courses trainees may attend | Written and practical assessment  repeated 3-4 months later  If trainees failed this assessment they were offered refresher teaching  9/33 passed the knowledge test |
| Wylie(50) | 2013 | 4 trainees  Clinicians and nurses | No formal assessment described | No specific requirements to ‘pass’ | 4 month post training follow up  All images recorded transferred to study supervisor for ongoing quality assessment and feedback.  No indication as to how these were assessed for quality. | No subsequent retesting described |

AC – Abdominal Circumference , ANC – Antenatal Clinic, BPD – Biparietal Diameter, CRL – Crown Rump Length, EDD – Estimated Date of Delivery, FL – Femur Length, HC – Head Circumference, OFD – Occipitofrontal Diameter, OSCE – Observed Structured Clinical Examination, TCD – Trans Cerebellar Diameter

*Table 5. Recommendations for the design and delivery of ultrasound training programmes presenting within the RE-AIM framework.*

| **Recommendation** | **Description of Recommendation** |
| --- | --- |
| **Reach** | *Providers*   - Who developed the training? - What are the qualifications/experience of those providing the training? - Which local stakeholders were involved in its organisation and delivery?   *Participants*   - Who participated in the training (demographic characteristics)? - How were they recruited? - Which individuals were included or excluded in the training? Why? - What proportion of eligible participants received the training? - What prior experience did they have? - What are their qualifications? - Were they given any incentive to participate? |
| **Effectiveness**  **or Efficacy** | - How were participants assessed and by whom? - What was the pass mark? How was this determined? - Describe what follow up was undertaken - Were trainees reassessed? - If reassessed what was the retention rate of skills/knowledge? - Were there any quality assurance processes? - Did the participants receive any formal certification or accreditation? If so, who bestowed this? |
| **Adoption** | *Setting level*   - Where was the training delivered? - Which sites were included or excluded in the intervention? Why? - Describe the characteristics of the participating sites - What site preparation was undertaken prior to the training?   *Individual level*   - What proportion of those invited to participate completed the training? - Describe individuals’ feedback on their experience of participating in the training |
| **Implementation** | *Content & Setting*   - Provide a brief description of the purpose of the training - Describe the learning objectives and how the training priorities were established - Describe the specific training materials provided to both the faculty and the participants and how these were developed   *Education Methodology*   - How was the training delivered? (lectures, small group sessions, ’hands on’ practice, level of direct supervision etc.) - What was the ratio of trainers to trainees? - Indicate how many ultrasound examinations were performed by each trainee and what proportion of these were directly supervised   *Fidelity*   - What percent of training delivery adhered to the original protocol? - Did the training require any adaptation or modification? If so, describe and explain the rationale for changes   *Costs*   - Who funded the training? - What was the final cost of the training? |
| **Maintenance** | - What consideration was given to factors affecting the delivery of the training? - What consideration was given to the ongoing provision of ultrasound and its integration into pre-existing services? - Were these studied formally?   *Individual level*   - What is the percentage of skills/knowledge retention amongst participants at or beyond 6months from original ultrasound training?   *Setting level*   - Is the program ongoing 6 months post formal study funding? - Has ultrasound training/provision been adapted into the local setting over time? |
